# Microvascular Changes during Viral Infections: A Systematic Review of Studies Using Retinal Vessel Diameter Assessments

**DOI:** 10.3390/biomedicines12071488

**Published:** 2024-07-05

**Authors:** Adam Saloň, Patrick De Boever, Nandu Goswami

**Affiliations:** 1 Division of Physiology & Pathophysiology, Otto Loewi Research Centre for Vascular Biology, Immunology, and Inflammation, Medical University of Graz, 8010 Graz, Austria; 2 Vascular Biology Center, Augusta University, Augusta, GA 30912, USA; 3 Faculty of Health and Social Sciences, Inland Norway University of Applied Sciences, 2624 Lillehammer, Norway; 4 Centre for Environmental Sciences, Hasselt University, 3500 Hasselt, Belgium; patrick.deboever@uza.be; 5 Antwerp University Hospital (UZA), 2650 Edegem, Belgium; 6 Center for Space and Aviation Health, College of Medicine, Mohammed Bin Rashid University of Medicine and Health Sciences, Dubai P.O. Box 505055, United Arab Emirates; 7 Integrative Health Department, Alma Mater Europaea, 2000 Maribor, Slovenia

**Keywords:** microcirculation, fundus imaging, SARS-CoV-2, HIV, retinal blood vessels

## Abstract

Viral infection frequently affects the cardiovascular system, and vascular disturbances in patients can lead to health complications. One essential component of the cardiovascular system that is vulnerable to the inflammatory effects of viral infections is the microcirculatory system. As a suitable and practical non-invasive method to assess the structure and function of the retinal microcirculation, a proxy for the microcirculatory system, retinal fundus imaging can be used. We examined the impact of viral infections on retinal vessel diameters and performed a systematic analysis of the literature. Our search was carried out on PubMed using predefined search queries. After a methodological filtering process, we were able to reduce the corpus of 363 publications to 16 studies that met the search parameters. We used a narrative review style to summarise the observations. Six studies covered COVID-19, seven described HIV, and three were included in the subgroup called others, covering viruses, such as Dengue Fever and Crimean–Congo Haemorrhagic Fever. Analysis of the literature showed that viral infections are associated with alterations in the retinal vessels’ vasoactivity. COVID-19 and other infections cause inflammation-associated the vasodilatation of microvasculature as a short-term effect of the infection. Long COVID-19 as well as HIV are the cause of chronic inflammation impacting microvascular morphology via retinal vessel diameter narrowing. The review emphasises the importance of the understudied area of viral infections’ effects on retinal microcirculation. Continuous research in this area is needed to further verify retinal fundus imaging as an innovative tool for the optimal diagnosis of microvascular changes. As changes in the microvasculature precede changes in bigger arteries, the early detection of microvascular changes can go a long way in reducing the morbidity and mortality associated with cardiovascular diseases.

## 1. Introduction

Viral infections have consequences beyond the virus’s direct impact. These include the effects of the infection itself, due to the virus replication, accompanying inflammation, and/or clinically manifesting fever, fatigue, or other clinical symptoms and signs. A complex and multifaceted—but important—research area is the exploration of the relationship between viral infections and their impact on cardiovascular health. This is clinically relevant, as viruses, such as influenza, human immunodeficiency virus (HIV), and the coronavirus SARS-CoV-2, can affect cardiovascular function and structure. These consequences may manifest as a direct viral invasion of cardiovascular tissues, harm brought on by inflammation, or the systemic sequalae (e.g., vasculitis, thrombosis, and endothelial dysfunction) of the viral infections, which directly affect the structure and function of the macro and microcirculatory blood vessels.

HIV continues to be one of the main challenges to the global public health system. It is responsible for approximately 40 million deaths, and despite yearly improvements in prevention, screening, and accessibility to antiretroviral treatment, its ongoing transmission increases the number of cases globally [1]. At the end of 2022, 39.0 million people were living with HIV, most of them in the African region [1]. The introduction of antiretroviral therapy made HIV a chronic condition rather than a fatal disorder. Although an antiretroviral therapy-caused adaptive immunity improvement is evident, a certain level of chronic inflammation persists. This permanent inflammation leads to endothelium activation and the attraction of monocytes and T-cells, followed by the activation of macrophages, which forms foam cells and leads to atheromatous lesion development. Earlier introduction of antiretroviral therapy has a higher potential to diminish the CD4+ and CD8+ T-cell disbalance [2]. Furthermore, HIV-positive individuals with advanced age show a higher rate of myocardial infarction, heart failure, and sudden cardiac death when compared to the general population [3,4,5]. Although new generations of antiretroviral drugs seem more beneficial to cardiovascular health than the old ones, they still pose uncertain cardiometabolic risks [6]. Therefore, the interconnection between HIV, antiretroviral therapy, and cardiovascular impacts represents the foundation for a lot of current research in this area [7,8,9,10,11].

In 2019, the world was shaken by the COVID-19 pandemic, caused by the severe acute respiratory syndrome coronavirus 2 (SARS-CoV-2). As of writing, COVID-19 has caused more than 700 million infections and almost 7 million deaths [12]. Although the main clinical demonstration of COVID-19 is pneumonia, the infection may unleash a cytokine storm with the overproduction of proinflammatory mediators and lead to multiorgan damage, frequently affecting the cardiovascular system. ACE2, the key receptor for virus internalization, is abundantly expressed in distinct tissues including the heart and vascular tissues [13,14]. Virus-mediated, ACE2-caused overactivation of the renin-angiotensin system triggers hypertension, congestive heart failure, and atherosclerosis [14]. While COVID-19 patients with cardiac comorbidities have a higher mortality rate, the infection itself poses a burden, increasing the prevalence of cardiovascular disorders such as myocardial injury, arrhythmias, acute coronary syndrome, and thromboembolism [15,16,17,18]. The endothelium inflammation and its long-lasting impact on microvasculature fitness lead to complete recovery around 3 months after the infection [19,20]. Furthermore, disrupted vascular function and higher arterial stiffness point to the impact of COVID-19 on larger arteries [21,22]. In addition, the virus can induce arrhythmias, myocarditis, valvular damage, and acute coronary syndrome [23].

Because of their negative effects on microcirculation—the network of tiny blood arteries that facilitates the flow of nutrients and waste products between blood and tissues—viral infections can induce physiological changes contributing to aggravating cardiovascular disease. An important aspect of the pathology of microcirculation during a viral infection is endothelial dysfunction, induced by inflammatory responses and cytokine productions. This endothelial dysfunction can lead to decreased vasodilation, increased permeability, and thrombosis. A direct viral invasion of endothelial cells might result in cellular damage or death, compromising the microcirculation’s integrity and functionality. The disturbance in microcirculatory function is implicated in the development of chronic diseases as well as in the progression and acute manifestations of cardiovascular disease.

An individual’s microvascular health can be assessed with a non-invasive analysis of the retinal microcirculation using fundus imaging or optical coherence tomography (OCT). The retinal vascular stems from the ophthalmic artery, an internal carotid artery branch. Alterations in the retinal microcirculation can also be a proxy for alterations in microcirculatory changes in other organs. Quantitative changes in retinal vessel parameters, such as the diameters of retinal veins and arteries, typically summarised in the literature as the Central Retinal Arteriolar Equivalent (CRAE) and Central Retinal Venular Equivalent (CRVE), are important indicators of the health and function of the retinal microcirculation, and changes in their diameters have been linked to various systemic diseases [24,25,26,27,28,29,30,31,32,33,34,35,36,37,38,39,40,41,42,43,44,45,46,47,48,49,50,51,52,53]. Studies have demonstrated that viral infections can alter the widths of retinal veins and arteries, resulting in structural and functional changes in the retinal vasculature [24,54,55,56,57,58,59,60,61,62,63,64,65,66,67,68].

The aim of this systematic review is to summarise the knowledge on the interaction between viral infections and retinal microcirculation, specifically the diameters of retinal veins and arteries in viral infections. The diameters of retinal microcirculation vessels have been chosen since they are considered promising metrics that are examined within numerous large cohort studies, and they are studied as a non-invasive marker of cardiovascular health [32,33,34,35,36,37,38,39,40,41,42,43,44,45,46,47,48,49,50,51,52,53]. A deeper understanding of the link between viral infections and retinal microcirculation could have substantial implications for the development of new diagnostic and therapeutic strategies for these diseases.

## 2. Methods

### 2.1. Identification and Protocol

A systematic review of the literature was conducted following the Preferred Reporting Items for Systematic Reviews and Meta-Analyses (PRISMA) Guidelines for Systematic Review [69]. We zoomed in on the topic to ensure an appropriate search strategy with search terms. This systematic review was registered at OSF REGISTRIES (https://osf.io/registries, accessed on 1 July 2024).

### 2.2. Eligibility Criteria

A comprehensive search of the literature, was performed using globally recognized scientific electronic databases. The generated reference list of the literature was manually verified for those articles that specifically investigated the effect of various viral infections on the diameters of retinal vessels via static retinal imaging. The review publications have been excluded from the search. This search was applied to the PubMed database. The latest search was performed on 28 January 2024.

The Population, Interest, Control, and Outcome (PICO) table used to define the present research criteria is shown in Table 1, along with the keywords. The population to be studied was chosen as “humans”; animal studies were excluded. Each article that underwent advanced validation by reading the whole text and did not include humans as participants of the study was excluded. For the clarity of the search strategy, the exclusion based on the publication type (such as “review”) was also added to this section. The intervention covers the keywords covering viral infections. The outcomes to be considered included retinal microcirculation diameter investigations, such as retinal arteriolar narrowing/dilatation, retinal venular narrowing/dilatation, and changes in retinal arterio-venular ratio. Furthermore, English language proficiency was established as a prerequisite for the inclusion of articles in subsequent consideration.

Participants of any age and sex were included. Full-length and abstract peer-reviewed articles were considered eligible for inclusion. The eligibility of articles was investigated in two rounds. In the first round, all retrieved records were screened by title and abstract, and each article was rated by the designation “relevant”, “irrelevant”, or “unsure” by AS and confirmed independently by NG and PDB. The retrieved records designed as “relevant” or “unsure” were fully read in the second round and the final selection was decided in consensus. The selected articles were included in this systematic review.

## 3. Results

**Figure 1:**
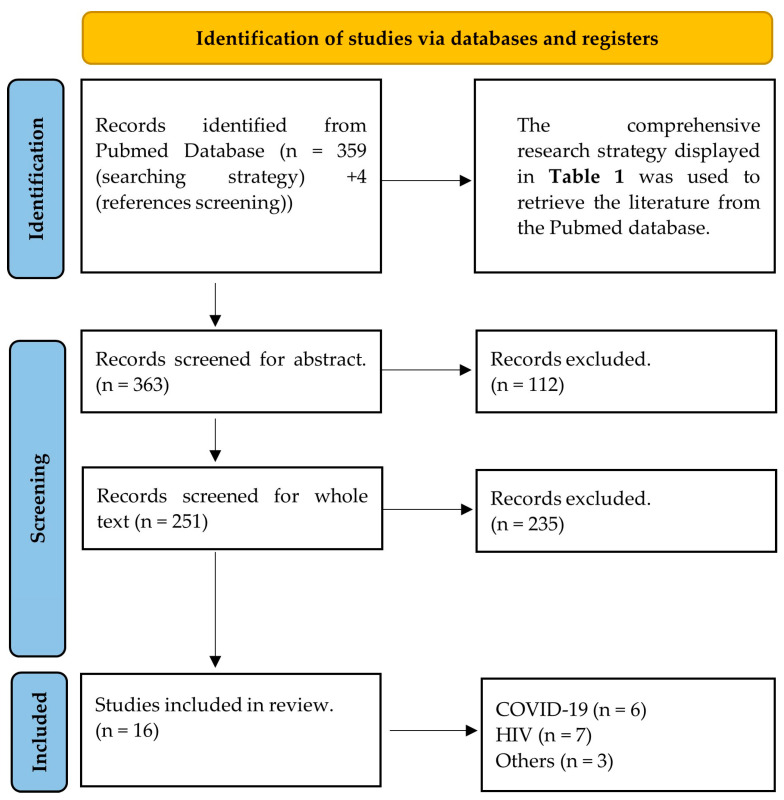
PRISMA 2020 flow diagram for systematic reviews that presents the search and screening strategy of the present study.

The systematic Pubmed search initially identified 363 research articles. The titles and abstracts of these were checked and 251 of them were retained. The whole texts of the selected articles were read and the final number of 16 was included in the study. The most common reason for papers to be excluded during the screening process was that even though they evaluated the effect of viral infections on the eye or retina, they did not cover specific evaluation of the diameters of retinal vessels. The majority of these papers covered topics including but not limited to central retinal artery/vein occlusion, macular oedema, or glaucoma, and did not employ vessel diameter evaluation, not even as a secondary outcome. These articles were beyond the initial scope of the original research question and were not further considered. The flow of the screening is displayed in Figure 1.

Sixteen papers were categorised into three groups based on the infection type: “COVID-19”, “HIV”, or “Other Viral Infections”, respectively “Others”. Table 2, Table 3 and Table 4 summarise the characteristics of these eligible studies. All 16 studies included in this review were published in English and contained original data. There were differences in study design (e.g., age of participants, sex, and duration of the infection), active/non-active infection (some studies focused on observing post-infection effects), and measurement intervals between the studies. Due to obvious heterogeneity between the studies as well as the limited number of papers, a meta-analysis was not performed. Therefore, we opted for a narrative review style.

## 4. Coronavirus Disease 2019

The six studies (two from Italy, two from Turkey, and one each from Germany and Slovenia) included in this section were published between 2020 and 2023 and included a total of 499 participants (217 COVID-19 patients and 282 control subjects) aged from 9 to 82 years [24,54,55,56,57,58] (Table 1). Five different imaging tools to capture static retinal images (Digital Retinography System fundus camera, optical coherence tomography (OCT), Spectralis OCT + HRA with Infrared Reflectance, Static Vessel Analyzer, and Retinal Camera Optomed Aurora) were used across the studies. The studies focused on retinal vasculature comparison either between patients and controls, patients over time of infection and after it, or investigated recovery after overcoming COVID-19. While there was a concurrence in the results of the COVID-19 effect on retinal vessel diameter between five studies, one study investigated the effect of post-COVID-19 syndrome with an opposing impact on the vessels.

The cross-sectional, Italian study SERPICO-19 investigated the presence of retinal alterations in patients with COVID-19 and subjects unexposed to the virus [54]. The study also examined the effect of infection severity on the retinal microcirculation profile. The study included individuals aged from 23 to 82 years (n = 54 COVID-19 patients; n = 133 unexposed subjects) measured at baseline and 6 months later [54]. The COVID-19 patients were aligned according to disease severity and the effect of severe and non-severe COVID-19 on CRAE and CRVE was tested at baseline and follow-up. The results showed that CRAE and CRVE at the baseline were significantly higher in COVID-19 patients compared to unexposed subjects [54]. Further analysis showed that while CRAE was positively associated with severe cases of COVID-19, CRVE showed a significant and positive association for both severe and non-severe cases compared to unexposed subjects. Additionally, a significant difference in CRVE between severe and non-severe cases with severe patients having a higher CRVE was shown. Thirty-three COVID-19 patients and sixty-four unexposed subjects agreed to join the follow-up study, out of which 32 and 53, respectively, had gradable fundus images both at baseline and 6 months later [55]. This report showed a significant reduction of 5.4% and 6.4% in CRAE and CRVE, respectively, in follow-up measurements [55]. Further analysis showed that both non-severe and severe COVID-19 patients had a higher CRAE and CRVE compared to unexposed subjects at baseline. This difference disappeared in non-severe cases at follow-up, but both CRAE and CRVE remained significantly higher in severe COVID-19 patients compared to unexposed subjects. The diameter differences between severe and non-severe COVID-19 patients were not noted.

The first study conducted in Turkey aimed to assess longitudinal changes in retinal vessel diameters [56]. The study included individuals aged from 9 to 78 years (n = 25 COVID-19 patients; n = 25 healthy controls) who were measured at baseline and at 4 months after remission. While the baseline diameters of the vessels in COVID-19 patients were increased compared to controls, their diameters decreased after remission and reached levels of controls. This agrees with the widening of retinal vessel diameters as an effect of COVID-19 [56].

Additionally, Gündoğan M and collaborators conducted a cross-sectional study in Turkey, between December 2020 and May 2021, to compare the differences in retinal vascular structure and choroidal thickness between the active disease and post-recovery periods in COVID-19 patients and healthy controls [57]. This study included 29–65 years old, 30 patients with severe COVID-19, and 30 age and sex-matched healthy controls [57]. They were measured at two different time points: active disease period (after positive polymerase chain reaction) and after recovery (three months after two negative PCRs). The results showed significantly higher thicknesses of vessel walls in the active disease period than after recovery, but not between after recovery and the control group [57]. The mean outer vessel diameters were higher in the active disease period than after recovery, but not between after recovery and the control group. The study confirmed the results above when it showed a COVID-19-mediated increase in retinal vessel diameters via the enlargement of the thicknesses of vessel walls. This effect was diminished by COVID-19 recovery.

A study conducted in Germany focused on endothelial dysfunction via evaluation of retinal microvasculature in patients with post-COVID-19 syndrome compared to healthy controls [58]. Furthermore, this study explored the potential of retinal microcirculation parameters as biomarkers for the diagnosis and management of patients with post-COVID-19 syndrome [58]. The study included individuals aged from 28 to 55 years; 41 patients with post-COVID-19 syndrome and 41 healthy controls. Out of the seven included studies in this section, this was the only one that did not focus directly on COVID-19 effects, but rather on long-lasting COVID-19 or post-COVID-19 syndrome and its impact on retinal vasculature. The study noted significantly narrower CRAE and lower retinal (arterio–venous ratio) AVR in patients with post-COVID-19 syndrome compared with healthy controls [58]. Additionally, when comparing post-COVID-19 syndrome patients with and without the encephalomyelitis/chronic fatigue syndrome, the ones with encephalomyelitis/chronic fatigue syndrome had significantly reduced CRAE and AVR. The authors investigated post-COVID-19 syndrome, perhaps the reason why the results oppose the findings of other studies in this section. Their results suggest that there are differences in the acute and chronic effects of COVID-19 on microvasculature, similarly, as pointed out and summarised by Hanssen and colleagues in diabetes [32].

The Slovenian study by Saloň and colleagues investigated the influence of COVID-19 on cardiovascular physiology and retinal microcirculation in 35 patients (aged from 50 to 70 years) recovering from COVID-19 infection [24]. The measurements were obtained at two time points: (1) the baseline measurements were collected, either on the day of hospital discharge if a negative PCR test was obtained, or on the 10th day after hospitalization if the PCR test was positive; (2) the second measurements were taken 60 days after hospitalization [24]. The COVID-19 recovery period showed significantly narrower CRVE and a trend in decreasing CRAE when compared with the baseline [24]. In case the baseline measurements, which were performed immediately after hospital discharge, when the effect of the infection is still distinct, patients are considered as COVID-19 patients, and the second measurements as healthy individuals, who could be considered as a control; moreover, the findings in this study agree with the others. However, it is important to say that this study did not have a control group; therefore, we cannot rely on control as hypothesised above.

## 5. Human Immunodeficiency Virus

The seven studies (two from the USA, two from Singapore, and one each from South Africa, Indonesia, and Turkey) included in this section were published between 2008 and 2022 and included a total of 2433 participants (1843 HIV patients and 590 control subjects) aged from 17 to 75 years [59,60,61,62,63,64,65] (Table 2). Six different imaging tools to capture static retinal images (Digital Fundus Camera (Zeiss FF-series), Wide-Angle Fundus Camera, 45° Retinal Camera with a digital camera back (10D SLR; Canon), Fundus Camera Canon CF-2, Nikon D70s, and Spectral-Domain OCT) were used across the studies. The studies focused on retinal vasculature comparison either between patients and controls, patients over time of infection, or investigated the associations with other risk factors, such as antiretroviral therapy and/or immunological biomarkers. Besides small discrepancies within the results of studies, the HIV, antiretroviral therapy, as well as viral load, reduced the diameter of arterioles and dilated the diameter of venules. Two out of seven included studies did not find any significant changes.

The first USA-based study investigated the possible connection between cocaine use and abnormalities in the retinal vasculature [59]. Even though the main aim of the study was not to zoom in on HIV infection and its reflection in vascular health, our study is relevant to include, as 42 out of 74 included participants (29–45 years) have been HIV positive [59]. The analysis did not reveal any connection between HIV and retinal microvascular parameters [59].

The study by Gangaputra et al., conducted in 2012, is the largest, with a sample size of n = 1250 [60]. The authors asked if there is a connection between HIV and the parameters of retinal vessels and how are these parameters interconnected with mortality [60]. They showed that smaller CRAE and larger CRVE were associated with a history of highly active antiretroviral therapy. The larger CRAE was associated with lower CD4+ T-lymphocyte count and worse health (Karnofsky scores) was strongly related to larger CRVE and smaller AVR [60]. Furthermore, wider CRVE and smaller AVR were strongly related to death on crude analyses. The study provided a lot of data related to HIV and the parameters of retinal microcirculation; however, no longitudinal changes over the HIV progression were observed; moreover, the study did not involve an HIV naive control group to compare with.

A case-control study conducted in Singapore included both HIV patients (n = 85) and healthy controls (n = 251) [61]. The study focused on revealing the differences in retinal microvascular parameters between these two groups. Additionally, it looked at the relationships between these parameters and blood-related HIV biomarkers [61]. Although no direct differences in retinal vascular calibres were observed between the groups, similarly to the previous study, it showed that increased viral loads in HIV patients were associated with lower CRAE and AVR [61].

The only study conducted in the African continent, where HIV is the most pronounced, was aimed at the relationship between the diameter of retinal vessels and clinical and demographic parameters by comparing HIV-infected individuals and healthy controls [62]. This study has a strong foundation in that it includes a control group accompanied by a respectable study sample size of n = 491, whereas other studies either did not include a control group or their sample size was significantly lower [62]. In HIV patients, compared with healthy controls, when values were not corrected, the results showed dilated and narrowed CRAE and CRVE, respectively [62]. Age as a factor narrowed the diameters of the vessels in HIV patients, which could at least partially explain the narrowing of CRVE seen in the unadjusted model. Potential factor/s or mechanism/s lying behind the dilatation of CRAE could be, for example, inflammation status and the associated impact on the endothelium. Age-adjusted CRAE, but not CRVE, was reduced with increasing duration of antiretroviral therapy and viral load in HIV patients. This effect of antiretroviral therapy confirmed the findings of the LSOCA study mentioned above. These results suggest that not only HIV alone but also antiretroviral therapy is a possible player in vascular damage that can be seen in retinal microcirculation.

Another study, as a part of the JakCCANDO (Jakarta, CMV, Cardiovascular, Antiretroviral, Neuropathy, Dental, Ophthalmology) project zoomed in on the interconnection of HIV, antiretroviral therapy, and cytomegalovirus concerning the retinal vasculature [63]. They included both HIV (n = 79) as well as control (n = 17) groups and measured them before antiretroviral therapy and in months 3, 6, and 12 [63]. The study showed that HIV patients had narrower retinal arterioles in the baseline as well as higher levels of cytomegalovirus antibodies [63]. The diameter of the retinal arteries decreased over twelve months of ART. Right arterial diameter correlated with cytomegalovirus antibodies and left arterial diameter in the third month, correlated with carotid Intima-Media Thickness. There was a negative correlation between left retinal arteriolar calibre at the 3rd or 12th month and CD4 T-cell counts at the 6th month. Furthermore, after adjustment for HIV RNA, smoking (dilatation) and alcohol (constriction) were the strongest predictors of retinal arteriolar calibre. Similarly to others, they showed that not only HIV itself leads to a reduction in retinal arteriolar diameter, but antiretroviral therapy does as well.

The cross-sectional study by Cetin et al. aimed to investigate changes in choroidal thickness in HIV-infected patients [64]. Secondly, the evaluations of retinal and vascular structural alterations, including diameters of the vessels, were performed. The healthy control group was included, and the gathered data were compared between the groups [64]. Even though they did not find any differences in retinal vascular diameters, the other results, such as retinal pigment epithelium being thinner in HIV-infected patients, or HIV-1 RNA being negatively correlated with choroidal thickness and positively correlated with retinal nerve fiber layer, showed the importance of retinal examination in HIV-infected patients [64].

The most recent report in this review section investigated vascular and immunological recovery in HIV patients with over 9 months of antiretroviral therapy [65]. The study included 100 HIV patients measured at baseline, and then at the 3rd, 6th, and 9th months. No control group was present [65]. CRAE increased over time in the study [65]. These observations oppose those in the study by Gangaputra et al., where extensive antiretroviral therapy reduced CRAE and increased CRVE. The report by Gangaputra et al. showed that patients with narrower CRAE and wider CRVE at baseline were significantly associated with 9-month reductions in CD4+ T-cell count, while their study presented that higher CRAE associates with lower CD4+ T-cells. This could be explained by different setups, sample sizes, populations, or stratification within these two studies. While Gangaputra is investigating a more general concept of the connection between HIV and retinal vascular parameters, this report focuses more on the effect of 9-month antiretroviral treatment.

## 6. Other Viral Infections

The three last studies (India, Turkey, and Australia) are included in the section other viral infections. One was published in 2006 and two others in 2021 (Table 3). They included 201 patients (Dengue Fever, Crimean–Congo Haemorrhagic Fever, or other infections) aged from 8 to 84 years [66,67,68]. No control subjects were included in these studies. Three different imaging tools (two published and one unpublished) to capture static retinal images (Slit-Lamp Biomicroscopy + Dilated Fundus Examination, and Non-Mydriatic Retinal Camera) were used across the studies. The studies examined the manifestation of Dengue Fever, Crimean–Congo Haemorrhagic Fever, or other infections relating to the health of the eye, especially the retina. Additionally, one study examined the effect of antibiotic treatment during various infections on retinal microcirculation. The three studies showed infection-related venular dilatation.

The first of the three studies included in the section “others” is the study that investigated ocular changes as an effect of dengue fever [66]. This study did not include a control group, and the methodology section as well as the method of ocular examination was not well explained. Even though only two (1.5%) patients presented these findings, the viral background of the study is a strong base for inclusion [66]. Furthermore, the laboratory findings, especially those related to haemorrhages, were measured. Fifty patients presented subconjunctival haemorrhage, and 84% of them had the characteristic petechial type of haemorrhage. Additionally, the study showed that thrombocytopenia was significantly associated with haemorrhages.

The second study is a prospective study evaluating the ophthalmologic examination of twenty-four children with Crimean–Congo Haemorrhagic Fever [67]. Two (8.3%) and seven (29.1%) children presented with dilatation of the retinal vein and tortuous retinal vessels, respectively [67]. Additionally, conjunctival hyperaemia was observed in 50% of patients. No changes in laboratory findings have been observed.

The last study in this section investigated retinal vessel diameters in acute infections before and after antibiotic treatment [68]. Patients with various infections such as respiratory, urinary, or skin infections, with an initial CRP level  >  100 mg/L, and CRP  <  100 mg/L after treatment, were included [68]. No healthy controls have been present in this study. The results showed decreased CRVE but no change in CRAE, in the patients compared to before and after antibiotics [68]. The observed change in CRVE correlated with initial white cell and neutrophil counts. Their results showed the infection-mediated impact of inflammation on vasculature seen in the retinal venules. However, the study did not present the source of the infections, and, therefore, bacterial, rather than viral infection, could be the reason for the antibiotic effect seen in their study.

## 7. Discussion

To the best of our knowledge, this is the first review investigating the relationship between viral infections and retinal microcirculation, with a focus on retinal vessel diameters. The possible alterations in vasoactivity associated with viral infections are highlighted by the current systematic review of 16 articles. The results imply that long-lasting COVID-19 and HIV are the cause of chronic inflammation, which is followed by effects on microvascular morphology, which reduces retinal vessel diameter. Short-term infections are causing inflammation-associated vasodilatation.

### 7.1. Viral Impact on Retinal Microvasculature

The summarised data of this systematic review are in almost complete agreement when talking about COVID-19 and its impact on retinal microvasculature. Four out of five studies investigating the direct effect of COVID-19 showed enlarged diameters of both arterioles and venules in the retina. One study did not investigate the effect of COVID-19 but post-COVID-19 syndrome (PCS) and recorded narrower retinal arterioles as the effect of PCS. Data suggest that while COVID-19-caused inflammation-associated vasodilatation of microvasculature is a short-term effect of the infection, PCS leads to opposite results and could pose the chronic effect of inflammation with an impact on microvascular morphology.

The research elucidating the HIV impact was more diverse in results. It is important to mention that it is hard to separate the other effects, especially the effect of antiretroviral therapy in this type of research, as the prevalence of recruited patients is an ongoing antiretroviral therapy or begins at the start of the study. This limitation explains the reason for the diversity in the results when most of them focus on the effect of antiretroviral therapy in HIV patients and not the direct effect of the disease.

Only two out of seven studies in this review present significant changes when compared to HIV patients with healthy controls. However, these independent studies present opposite results, which multiple accompanying factors, such as race, age, sex, or the duration of antiretroviral therapy, could explain. Overall, the data in this review indicate that viral load and antiretroviral therapy reduce the tone of retinal vessels, especially arterioles. This could be explained by increased ROS levels as an effect of HIV-related inflammation followed by reduced NO bioavailability. The relationship between CD4+ count and retinal microcirculation in HIV could support these suggestions. However, although some studies present the correlation between retinal microcirculation parameters and CD4+, the results are contradictory, and more investigation must be performed in this area. Additionally, two out of seven studies dealing with the effect of HIV on retinal microcirculation did not present significant findings related to any of the investigated retinal microcirculation parameters.

While the previous lines have shown that research dealing with COVID-19 and HIV infections is relatively abundant, research on other viral infections concerning retinal microcirculation lags. However, it is important to say that while the previously mentioned affects millions of people globally, most other infections are not as widespread and/or are easily overcome by the immune system. 

While all three studies suggest that infections lead to vasodilation, one also adds that antibiotics can reverse this effect, particularly in retinal venules. However, the study presenting the effect of antibiotics did not present the source of the infections, and, therefore, bacterial, rather than viral infection, could be the reason for the antibiotic effect seen in their study. Additionally, the observed retinal vessel dilatation may be explained by the inflammation caused by viral infection. The lack of research highlighted in the present review, especially in this paragraph, should motivate future investigations to supplement this area of research.

### 7.2. Viral Infections and Inflammation: Impacts on Vascular Stability

Viral infections and the inflammation associated with them are the driving engines of vascular instability. Elevated inflammation mediators and recruiting immune cells increase inflammation in a positive loop, and its extension may lead to endothelial dysfunction. Oxide-redox disbalance, increase in ROS, and its scavenging of NO to peroxynitrite production reduce NO bioavailability and are the first steps to endothelial dysfunction and later atheroma formation. Microvasculature is an important nourishment delivery network to end organs and diverter of metabolic waste products. Inflammation-associated vascular impartment may affect these important functions and the onset of organ damage. The inflammation-induced vascular vasodilatation to increase the flow and permeability of the endothelium to facilitate immune cell infiltration, leading to virus elimination and healing process initiation, are typical acute responses of the body to infection. However, if the inflammation persists for a long time, acute inflammation continuously turns chronic. Chronic inflammation leads to irreversible morphological and functional changes, such as endothelial-to-mesenchymal transition [70] and endothelial dysfunction, thereby losing the vasoactivity of vessels.

### 7.3. Methodological Discrepancies in Retinal Microvasculature Assessment

In considering the calculation of CRAE and CRVE, it is important to mention the methodological discrepancies among studies. For instance, within COVID-19 studies, five different imaging tools to capture and four different types of software to analyse—Mona-REVA [24], ARIA, an open-source software developed on the MATLAB platform [54,55], Spectralis [56,57], and Vesselmap 2^®^ [58]—were employed. Likewise, studies investigating HIV utilised six various imaging tools and four various software applications—IVAN [59,60,62], Image J [63], SIVA [61,65], and SD-OCT software [64]. The remaining three studies utilised at least two different imaging tools, with only one study specifying the use of IVAN software [68]. Therefore, the variations between the results within the studies could stem from differences in camera resolutions and the software packages used for image analysis. Additionally, each software or grader can use the different regions within the fundus image to analyse vessels, and the approaches to vessel selection may vary in terms of the number of vessels used for CRAE and CRVE calculation. Such technical differences may result in variations in sensitivity and absolute calculations of CRAE/CRVE. Therefore, when interpreting and comparing CRAE/CRVE values across studies, it is important to consider these methodological differences to ensure accurate and reliable interpretation.

One important consideration in interpreting our findings is the potential influence of treatments administered during viral infections. For instance, corticosteroids, used in the management of COVID-19, can have significant effects on microcirculation. However, corticosteroids are only given to patients with severe or critical COVID-19 [71].

While our review highlights changes in retinal vascular diameter during acute viral infections, it is crucial to acknowledge that these changes may not be attributable solely to the direct effects of the virus. Any observed response of the microcirculation is the result of a complex interplay by a multitude of external triggers and internal physiological processes. Medical interventions to abate an infection may also have a direct impact on retinal vessel changes. Future clinical studies should try to disentangle the effects of medication and infection as the studies retrieved in the current review did not include enough details to do so.

### 7.4. Retinal Vessel Diameters as Biomarkers of Health Outcomes

The interplay between the diameters of retinal vessels and different health outcomes has been under comprehensive investigation in multiple cohort studies. Multiple of these studies demonstrated the association between retinal vessel diameters and the development of hypertension.

While several large studies, including Atherosclerosis Risk in Communities (ARIC) study [33], the Multi-Ethnic Study of Atherosclerosis (MESA) [34], the Blue Mountains Eye Study (BMES) [35], the Beaver Dam Eye Study (BDES) [36], and the Rotterdam Study (RS) [37], unanimously showed an association between narrower retinal arterioles and incidence of hypertension, the contribution of changes in venular diameter noted only in the MESA and RS differed. Similarly to the suggestion of the present systematic review, chronic inflammation followed by vascular impairment and narrowing of retinal arterioles could be the precursor of later hypertension observed in those cohort studies.

Moreover, while the BMES study showed, that higher CRVE has been linked to obesity and significant weight gain [38], the ARIC study extended findings to metabolic disorders when it showed reduced CRAE and enlarged CRVE in individuals with the metabolic syndrome [39]. In addition, obesity, as a systemic chronic inflammatory disease, is associated with enlarged CRVE [40]. The results summarised by the present systematic analysis and its suggestion of retinal arteriolar narrowing as an effect of chronic inflammation are partially in concordance with those.

Interestingly, the interconnection between retinal vessel diameters and diabetes seems to vary based on the study design. While cross-sectional analyses showed associations of diabetes prevalence with wider arterioles [41,42,43,72], prospective analyses presented associations between the risk of developing diabetes and arteriolar narrowing [44,45,46]. This may suggest an intricate interplay between retinal vessel biology and metabolic health over time, as well as differences in the acute and chronic impacts of diabetes, as systemic inflammation disease, in retinal vessels similarly proposed by the present review. Additionally, a cross-sectional Maastricht study established the association between higher HbA1c and the prevalence of diabetes with wider retinal arterioles [47].

On top of that, several cohort studies such as the Cardiovascular Health Study (CHS), BDES, BMES, and ARIC showed an association of both arteriolar narrowing and venular widening with incident coronary artery disease [48,49,50]. Furthermore, narrower arterioles and wider venules have been linked to an increased risk of heart failure, stroke, and mortality in various cohort studies [51,52,53]. This underscores the prognostic significance of retinal vessel morphology in cardiovascular health. 

Moreover, changes in retinal vessel diameters were also investigated as promising predictors of stroke incidence. A meta-analysis of six previously mentioned cohort studies, namely, ARIC, BDES, BMES, Rotterdam Study, CHS, and AusDiab, presented that wider venular diameters are associated with a higher risk of stroke (20 μm increase in venular diameter was associated with a 15% higher risk of stroke) [48]. No significant associations with arteriolar diameter were observed.

Overall, the evidence from the above-discussed globally performed cohort studies suggests that retinal vessel diameters may serve as valuable biomarkers of different health outcomes. However, it is important to distinguish between temporary vascular changes observed during acute viral infections and more permanent changes associated with chronic cardiovascular disorders. Continued physiological challenges and lifestyle effects are known to induce structural changes in retinal vessel widths and increase the risk and incidence of cardiometabolic diseases. Multiple studies have also documented that retinal vessel widths, as measured on repeated fundus images, change in response to shorter exposures of risk factors such as diet [73], exercise [74,75,76], and air pollution exposure [77,78]. These latter observations are in line with the observations made during periods of viral infection. Individual retinal responses may help to predict infection-related vascular susceptibility, response to therapy, or the post-infection recovery process. We suggest that these topics be investigated in more dedicated study setups to help unravel the underlying pathophysiological mechanisms of these conditions concerning retinal vasculature and optimise the clinical utility of retinal vessel assessment in risk stratification and preventive care.

## 8. Summary

Viral infections can significantly impact the cardiovascular system, resulting in vascular disturbances and health complications. The microcirculatory system is receptive to the inflammatory effects of viral infections. Fundus imaging is a valuable non-invasive tool for assessing the structure and function of the retinal microcirculation, a proxy for the comprehensive microcirculatory system. However, we have a limited understanding of how viral infections affect retinal microcirculation. Our systematic analysis of 16 relevant studies, covering various viruses including COVID-19, HIV, and others, suggests retinal vessel vasoactivity changes associated with viral infections. While acute viral infections may induce short-term inflammation-associated vasodilation, chronic infections can cause persistent inflammation followed by retinal vessel diameter reduction. This work highlights the importance of further research in understanding the influence of viral infections on retinal microcirculation. Future investigation may unravel pivotal reveals to improve diagnostic accuracy, guide therapeutic strategies, and mitigate complications associated with viral-induced microcirculation changes.

## 9. Limitations

The potential confounding effect of medications is a major limitation. Currently, there is no possibility to disentangle between direct viral effects and additional effects of administered medication. Future clinical studies should try to uncover the effects of medication and infection itself because the studies retrieved in the context of the current review did not include enough details for doing this. The lack of methodological information in the retrieved studies including the use of masked graders and the assessment of reproducibility between graders poses another limitation. Furthermore, the used types of software packages and procedures to analyse retinal images differed between the studies. This makes it difficult to compare absolute retinal vessel width changes. However, as previously published, physiological trends and retinal vessel observations are reliable independent of the used software packages and analysis procedures because CRAE and CRVE are robust metrics [79,80]. Future research studies could enhance transparency and rigour by providing comprehensive details on these methodological aspects. Finally, the long-term impact of the observed microvascular changes remains uncertain. Risk factors (such as air pollution) have both short-term as well as long-term cardiovascular effects. We see a similarity between the short-term response to infection and a potential systemic effect. While our findings provide valuable insights, the clinical significance of these changes is not yet clear and requires further investigation. Despite these limitations, our review offers important insights into the impact of viral infections on retinal vascular diameters and provides a foundation for future research to build upon.

## 10. Conclusions and Future Directions

Our systematic review sheds light on the potential impact of viral infections on retinal microcirculation. The reviewed data suggest that while acute infection and related inflammation, as seen in COVID-19, may be associated with short-term vasodilation, chronic conditions such as long-COVID HIV could contribute to persistent inflammation and potential vessel diameter reduction. Confirmation of these hypotheses is warranted to improve diagnostics and therapeutic strategies in viral infections-related microvascular health complications.

## Figures and Tables

**Table 1:** Displayed are Population, Interest, Control, and Outcome (PICO), used to define the research criteria, the keywords used to retrieve the literature from the Pubmed database, and the search strategy.

Category	Specific Category	Keywords	Strategy
Population	Inclusion and Exclusion Criteria	(“Animals” [Mesh] NOT (“Animals” [Mesh] AND “Humans” [Mesh]))	#1
		review [Publication Type] OR “systematic review” [Publication Type] OR “systematic literature review” [Publication Type] OR “meta-analysis” [Publication Type] OR “meta analysis” [Publication Type] OR “meta-analytic review” [Publication Type]	#2
		Search #1 OR #2	#3
Interest	Viral Infections	“viruses” [Mesh] OR Virus* [tiab] OR “viral particle*” [tiab] OR “enteric virus*” [tiab] OR “viral contamination*” [tiab]	#4
Control	N/A	N/A	
Outcome	Retinal Microcirculation Diameter Investigations	“retinal vessels” [Mesh] OR “retinal vessel*“ [tiab] OR “retinal vessel diameter*“ [tiab] OR “retinal arter*“ [tiab] OR “retinal vein*“ [tiab] OR “retinal vascular calibre*“ [tiab] OR “retinal vascular caliber*“ [tiab] OR “retinal arteriolar calibre*“ [tiab] OR “retinal arteriolar caliber“ [tiab] OR “retinal venular calibre*“ [tiab] OR “retinal venular caliber*“ [tiab] OR “arteriovenous ratio*“ [tiab] OR “retinal arteriolar dilatation*“ [tiab] OR “retinal venular dilatation*“ [tiab] OR “retinal vascular change*“ [tiab] OR “retinal blood vessel*“ [tiab] OR “arteriolar narrowing*“ [tiab] OR “retinal arteriolar narrowing*“ [tiab] OR “retinal microcirculation*“ [ tiab] OR “retinal vasculature*“ [tiab]	#5
**Search strategy**		#4 AND #5 NOT #3	

**Table 2:** **Coronavirus disease 2019.** The table includes the manuscripts of the subsection Coronavirus disease 2019.

Title	Study Details	Study Design	Target Group	Imaging Tool	Purpose	Summary of Findings	
**Coronavirus Disease 2019 (COVID-19)**							
Retinal findings in patients with COVID-19: Results from the SERPICO-19 study	Alessandro Invernizzi et al. (2020), Italy [54]	Cross-sectional (SERPICO-19)	23–82 years (n = 54 + 133; COVID-19 patients + unexposed subjects)	Digital Retinography System (DRS) fundus camera (CenterVue, Padua, Italy)	The investigation of the presence of retinal alterations in patients with COVID-19 and subjects unexposed to the virus by using fundus photographs.	Diameters of both, arteries, and veins were higher in COVID-19 patients compared to unexposed subjects. The diameter of veins was positively associated with COVID-19 both in severe and non-severe cases compared to unexposed subjects. Moreover, the diameter of the veins in COVID-19 patients negatively correlated with the time from symptom onset and positively correlated with disease severity.	COVID-19: ↑ CRAE; ↑ CRVE
Retinal vessels modifications in acute and post-COVID-19	Alessandro Invernizzi et al. (2021), Italy [55]	Cross-sectional, longitudinal study (SERPICO-19)	24–72 years (n = 32 + 53; COVID-19 patients + unexposed subjects)	Digital Retinography System (DRS) fundus camera (CenterVue, Padua, Italy)	Investigation (at baseline and 6 months later) of alterations of the retina and its vasculature in patients with COVID-19 within 30 days of onset of symptoms.	At baseline, arteriolar and venular diameters were significantly higher in COVID-19 patients compared to unexposed subjects. Both significantly decreased in COVID-19 patients at follow-up. Vessel diameter remained significantly higher in severe COVID-19 patients compared to unexposed subjects after 6 months.	COVID-19: ↑ CRAE; ↑ CRVE
Retinal Vessel Diameter Changes in COVID-19 Infected Patients	Nazife Aşıkgarip et al. (2021), Turkey [56]	Prospective study	9–78 years, (n = 25 + 25; COVID-19 patients + healthy controls)	Optical coherence tomography (OCT)	To assess longitudinal changes of retinal vessel diameters measured in patients with COVID-19.	While the baseline diameters of the vessels in COVID-19 patients were increased compared to controls, their diameters decreased after remission in all quadrants in comparison to baseline measurements.	COVID-19: ↑ CRAE; ↑ CRVE
Change in retinal vessel diameter and choroidal thickness in patients with severe COVID-19: Change In Retinal Parameters In Patients With Severe COVID-19	Gündoğan M et al. (2022), Turkey [57]	Prospective, cross-sectional study	29–65 years (n = 30 + 30; COVID-19 patients + healthy controls)	Spectralis OCT+HRA with infrared reflectance (IR) images	To compare the differences in retinal vascular structure and choroidal thickness between the active disease and post-recovery periods in COVID-19 patients and healthy controls.	The study did not find changes in lumen diameter in either artery or vein.	-
Persistent endothelial dysfunction in post-COVID-19 syndrome and its associations with symptom severity and chronic inflammation	T. Kuchler et al. (2023), Germany [58]	Observational prospective cohort study (“All Eyes on PCS”)	41 COVID-19 patients (42.2 y ± 12.2) and 41 healthy controls (41.8 y ± 13.7)	Static Vessel Analyzer (IMEDOS Systems, Jena, Germany based on TRC-NW8 non-mydriatic retinal camera; Topcon, Tokyo, Japan)	Investigation of the endothelial function through evaluation of retinal microcirculation in patients with post-COVID-19 syndrome (PCS) compared to an age- and gender-matched healthy cohort.	Narrower central retinal artery equivalent (CRAE; 178.1 [167.5–190.2] vs. 189.1 [179.4–197.2], *p* = 0.01) and lower arteriolar–venular ratio (AVR; (0.84 [0.8–0.9] vs. 0.88 [0.8–0.9], *p* = 0.007) were observed. Additionally, significantly reduced CRAE (183.5 [177.4–197.0] vs. 174.0 [161.5–181.0], *p* = 0.03) and AVR (0.88 [0.82–0.91] vs. 0.82 [0.77–0.86], *p* = 0.02) in PCS patients with CFS were observed.	PCS: ↓ CRAE; ↓ AVR
A pilot study: Exploring the influence of COVID-19 on cardiovascular physiology and retinal microcirculation	A. Saloň et al. (2023), Slovenia [24]	Pilot, longitudinal study, as a part of a larger project	35 COVID-19 patients (60 ± 10 years)	Retinal camera Optomed Aurora (Optomed Oy, Oulu, Finland)	Assessment of cardiovascular changes in patients post-COVID-19 hospital discharge, examining both microvascular and macrovascular parameters. Measurements were taken at two post-discharge time points: on the day of discharge or day 10 post-hospitalization and 60 days post-hospitalization.	A significantly narrower CRVE (from 240.94 μm, SD: 16.05, to 198.05 μm, SD: 17.36, p = 0.013) and trend of decreasing CRAE (from 138.87 μm, SD: 12.19, to 136.77 μm, SD: 13.19, p = 0.068) were recorded when two measurement time points have been compared.	COVID-19: ↑ CRAE; ↑ CRVE

**Table 3:** **Human immunodeficiency virus.** The table includes the manuscripts of the subsection human immunodeficiency virus.

Title	Study Details	Study Design	Target Group	Imaging Tool	Purpose	Summary of Findings	
HIV (Human Immunodeficiency Virus)							
Early retinal vascular abnormalities in African-American cocaine users	Ivan Y-F Leung (2008), USA [59]	Population-based cross-sectional study	29–45 years (n = 68, out of which 42 HIV-positive patients)	Digital fundus camera (Zeiss FF-series)	Examination of the potential association between cocaine use and early retinal vascular abnormalities.	No significant associations were observed between HIV infection and any retinal vascular parameters.	-
Retinal vessel caliber among people with acquired immunodeficiency syndrome: relationships with disease-associated factors and mortality	Sapna Gangaputra et al. (2012), USA [60]	Longitudinal Study of the Ocular Complications of AIDS (LSOCA)	38–48 years (n = 1250; HIV-positive patients)	Wide-angle fundus camera	Assessment of the associations between retinal vessel calibers, factors related to AIDS, and mortality.	Narrower retinal arterioles and venules were related to a history of highly active antiretroviral therapy (ART); and larger CRAE with lower CD4+ T-lymphocyte count. There was a 12% increase in mortality risk per quartile of decreasing AVR.	↑ HIV ART: ↓ CRAE; ↓ CRVE, ↑ CRAE: ↓ CD4+, ↓ AVR: ↑ Mortality
Retinal vascular parameter variations in patients with human immunodeficiency virus	Petrina B. Tan et al. (2013), Singapore [61]	Case-control study (SEED program)	26–64 years (n = 85 + 251; HIV-positive patients + healthy controls)	45° retinal camera (Canon CR-DGi; Canon, Tokyo, Japan) with a digital camera back (10D SLR; Canon)	The study compares the retinal vascular parameters in patients with HIV infection with healthy controls and determines the relationship between these parameters and HIV-related blood biomarkers.	No direct differences in retinal vascular calibers were observed between the groups. Increased viral loads in HIV patients were associated with decreased retinal arteriolar caliber and decreased arteriolar-venular ratio.	↑ viral load: ↓ CRAE and ↓ AVR
Retinal arterioles narrow with increasing duration of anti-retroviral therapy in HIV infection: a novel estimator of vascular risk in HIV?	Sophia Pathai et al. (2013), South Africa [62]	Case-control study	35–48 years (n = 242 + 249; HIV-positive patients + healthy controls)	Fundus camera Canon CF-2	Investigation of the relationship between retinal vessel calibers and clinical and demographic characteristics in HIV-infected individuals in South Africa.	Unadjusted arteriolar diameters tended to widen and unadjusted venular diameters tended to narrow in HIV patients compared with healthy controls. Age as a factor modified diameters of vessels; narrower diameters in HIV patients but not in healthy controls. In HIV patients, retinal arteriolar diameters narrowed with increasing duration of HIV ART, independently of age, and with an HIV viral load >10,000 copies/mL while on HIV ART. HIV-related venular changes were not detected.	HIV: ↑ CRAE and ↓ CRVE, ↑ HIV ART duration and ↑ HIV viral load: ↓ CRAE
Factors Affecting the Health of Retinal Vessels in Human Immunodeficiency Virus Patients Beginning Anti-Retroviral Therapy	Lukman Edwar et al. (2019), Indonesia [63]	Comprehensive, longitudinal study (JakCCANDO)	19–48 years (n = 79 + 17; HIV-positive patients + healthy controls)	Nikon D70s (Tokyo, Japan) 6 megapixel camera	The study assesses the effects of HIV, ART, and cytomegalovirus (CMV) on the diameter of retinal arteries, as a non-invasive approach to measure vasculopathy in HIV patients beginning ART.	Patients with HIV had narrower retinal arteries and higher levels of CMV antibodies than healthy controls. The diameter of the retinal arteries decreased over twelve months of ART. Right arterial diameter correlated with CMV antibodies and left arterial diameter at 3rd month, correlated with carotid Intima-Media Thickness (cIMT). Smoking and alcohol consumption were the strongest predictors of retinal arterial diameter after adjustment for HIV RNA. While the diameters of patients who confirmed smoking were wider compared to non-smokers, those who confirmed alcohol consumption were smaller.	HIV: ↓ CRAE, ↑ HIV ART duration: ↓ CRAE
THE THICKNESSES OF CHOROID, MACULAR SEGMENTS, PERIPAPILLARY RETINAL NERVE FIBER LAYER, AND RETINAL VASCULAR CALIBER IN HIV-1-INFECTED PATIENTS WITHOUT INFECTIOUS RETINITIS	Cetin, Ebru N. et al. (2019), Turkey [64]	Cross-sectional study	17–75 years (n = 45 + 47; HIV-positive patients + healthy controls)	Spectral-domain optical coherence tomography (SD-OCT)	To evaluate choroidal, macular, and peripapillary retinal nerve fiber layer thicknesses and retinal vascular caliber alterations in HIV-1–infected patients without opportunistic infections.	The differences in retinal vascular caliber were not significant between the groups.	-
Brief Report: Retinal Microvasculature and Immune Restoration Among South Eastern Asian Patients With HIV/AIDS Over a 9-Month Antiretroviral Therapy	Li, Ling-Jun et al. (2022), Singapore [65]	Prospective cohort study	45.6 (SD 10.2) years (n = 100; HIV)	45° retinal camera (Canon CR-DGi; Canon, Tokyo, Japan) with a digital camera back (10D SLR; Canon)	This study aims to investigate whether retinal vascular abnormalities, indicative of real-time immune dysfunction, correlate with immune restoration in 100 HIV/AIDS patients undergoing a 9-month ART regimen.	Narrower arteriolar caliber (per 10 μm decrease), and wider venular caliber (per 10 μm increase) in the retina assessed at baseline were significantly associated with 9-month reductions in CD4+ T-cell count by 52.97 cells/μL (P = 0.006) and 33.55 cells/μL (P = 0.01) accordingly.	↓ CRAE and ↑ CRVE: ↓ CD4+

**Table 4:** **Other viral infections.** The table includes the manuscripts of the subsection Other Viral Infections.

Title	Study Details	Study Design	Target Group	Imaging Tool	Purpose	Summary of Findings
Other Viral Infections						
Ocular manifestations of dengue fever in an East Indian epidemic	Harpreet K Kapoor et al. (2006), India [66]	Observational study	13–65 years (n = 134; dengue fever)	-	To document the range of ocular manifestations observed in patients with dengue fever and to determine if there are any notable associations with specific laboratory parameters.	Two (1.5%) patients had dilatation and tortuosity of vessels as the only finding in both eyes.
Increased Retinal Vessel Tortuosity Associated with Crimean-Congo Hemorrhagic Fever in Children	Duygu Yalinbas et al. (2021), Turkey [67]	Prospective study	12.4 ± 3.6 years (n = 24; Crimean-Congo Hemorrhagic Fever)	Slit-lamp biomicroscopy, and dilated fundus examination	Children diagnosed with Crimean–Congo Hemorrhagic Fever underwent a complete ophthalmologic examination.	The fundus examination showed, that two (8.3%), out of twenty-four children, were found with dilatation of the retinal vein.
Increased retinal venular calibre in acute infections	Cara Fitt et al. (2021), Australia [68]	Observational study	65.7 ± 18.4 years (n = 43; infections)	Non-mydriatic retinal camera (Canon CR5-45, Tokyo)	To investigate the effect of acute infections (participants with infections and elevated CRP levels (>100 mg/L)) on retinal arteriolar and venular calibres, and to determine whether changes in calibres occur as infections resolve (before and after antibiotic treatment). Additionally, the study aims to explore the relationship between changes in retinal vessel calibre and inflammatory markers, particularly CRP levels.	The mean venular calibre (CRVE) of participants decreased from 240.9 ± 26.9 μm to 233.4 ± 23.5 μm (*p* = 0.0017).

## Data Availability

No new data were created or analysed in this study. Data sharing is not applicable to this article.
